# A case report of a rapidly growing giant coronary pseudoaneurysm: challenges and lessons learned

**DOI:** 10.1093/ehjcr/ytad637

**Published:** 2024-01-03

**Authors:** Hani J Alturkmani, Srikanth Vallurupalli, Malek Al-Hawwas, Kristofer T Freeland, Barry F Uretsky

**Affiliations:** University of Arkansas for Medical Sciences, 4301 West Markham Street, Little Rock, AR 72205, USA; University of Arkansas for Medical Sciences, 4301 West Markham Street, Little Rock, AR 72205, USA; Central Arkansas Veterans Healthcare System, Cardiology Section, 4300 West Seventh Street, 5C-100, Little Rock, AR 72205, USA; University of Arkansas for Medical Sciences, 4301 West Markham Street, Little Rock, AR 72205, USA; Central Arkansas Veterans Healthcare System, Cardiology Section, 4300 West Seventh Street, 5C-100, Little Rock, AR 72205, USA; Department of Cardiac Surgery, Arkansas Heart Hospital, 1701 S Shackelford Road, Little Rock, AR 72211, USA; University of Arkansas for Medical Sciences, 4301 West Markham Street, Little Rock, AR 72205, USA; Central Arkansas Veterans Healthcare System, Cardiology Section, 4300 West Seventh Street, 5C-100, Little Rock, AR 72205, USA

**Keywords:** Percutaneous coronary intervention, Coronary stenting, Cardiac surgery, Coronary pseudoaneurysm, Case report

## Abstract

**Background:**

Coronary pseudoaneurysm is a rare, potentially fatal, complication of coronary intervention. A challenging management case of a giant right coronary pseudoaneurysm is presented.

**Case summary:**

A 56-year-old man presented with an atypical presentation for ST-elevation myocardial infarction. Initial angiogram showed a crescent-shaped ostial lesion with probable connection to the aorta, which disappeared after placing a drug-eluting stent. A few hours later, patient was found to have staph aureus bacteraemia and infective endocarditis for which he received a prolonged antibiotic course. Patient presented a few weeks later with second degree heart block. Echocardiography showed a large cystic lesion adjacent to the right coronary cusp suspicious for a coronary pseudoaneurysm, which was confirmed with angiography. Attempts to treat it with a covered stent were unsuccessful and patient ultimately underwent surgical resection.

**Discussion:**

Coronary pseudoaneurysm develops when there is a contained breach of all three layers of the vessel. It may develop from direct iatrogenic trauma to the vessel wall but can be infectious in aetiology. The treatment approach remains uncertain due to limited evidence. Here, we present the diagnostic and technical challenges of managing such an uncommon entity and discuss an algorithm for management.

**Learning points**

1. Management of giant coronary pseudoaneurysm should be individualized using a Heart Team approach.
2. ‘Unexpected’ technical issues should be anticipated with alternate plans prepared should they arise.

## Introduction

Coronary artery pseudoaneurysm is a rare, potentially fatal, complication of stenting.^1^ While most interventional cardiologists have encountered on occasion this condition in practice, optimal management remains unclear due to its rarity without guidelines to aid in management. The literature on pseudoaneurysm management is scant and comprised predominantly of case reports. When the pseudoaneurysm compromises haemodynamics such as with rupture into the pericardium with ensuing tamponade, emergency surgery is usually recommended.^1^ However, when a small asymptomatic pseudoaneurysm is discovered incidentally, the appropriate approach is less certain and has been treated by exclusion with a covered stent or coils, surgical excision, or ‘watchful waiting’ with periodic imaging surveillance.^1^ Here, we present a challenging case of a giant right coronary artery (RCA) pseudoaneurysm and its management.

## Summary figure

IV, intravenous; LV, left ventricular; PCI, percutaneous coronary intervention; RCA, right coronary artery; STEMI, ST-segment elevation myocardial infarction; TEE, transoesophageal echocardiogram.

**Figure:**
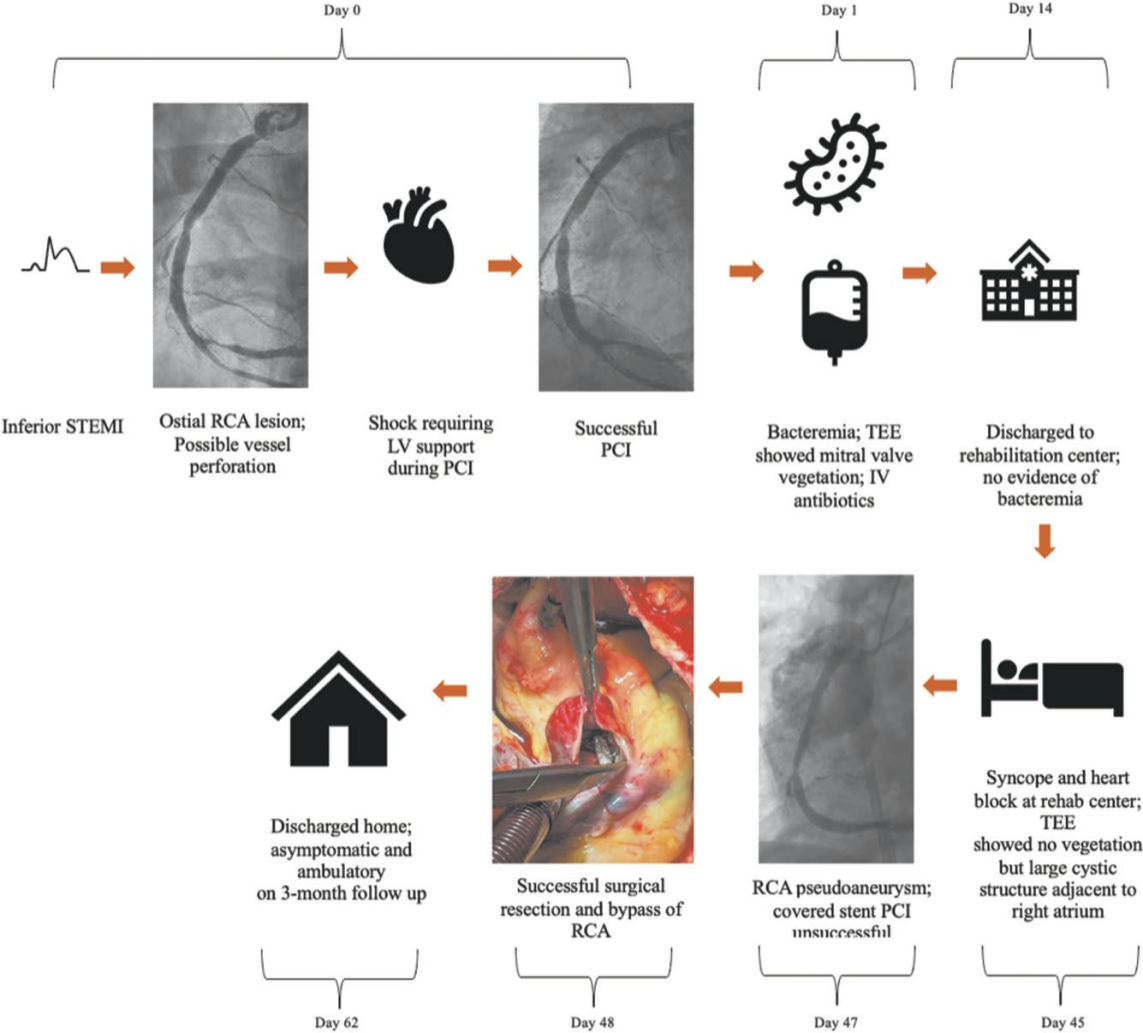


## Case presentation

**Figure 1:**
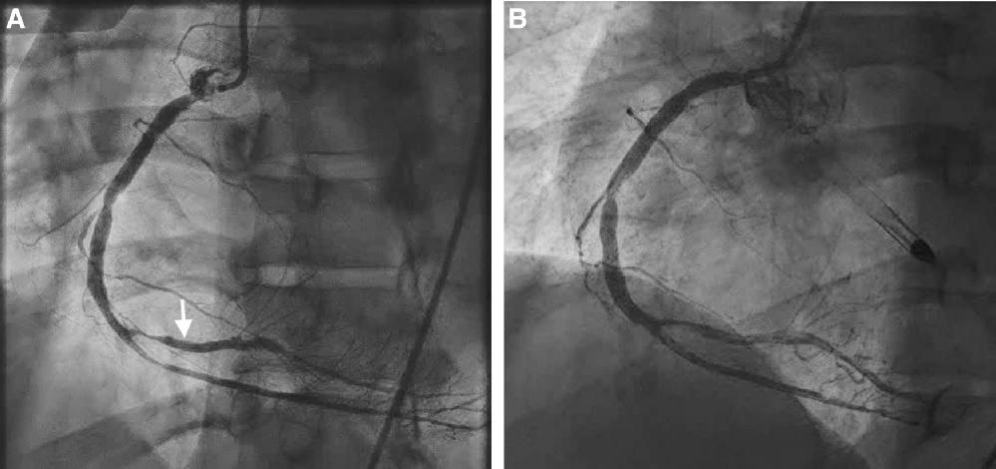
Initial presentation angiogram: *A*) left anterior oblique view of right coronary artery pre-drug-eluting stent implantation showing crescent-shape dye collection at site of near-total occlusion of the proximal right coronary artery; *B*) post-drug-eluting stent implantation shows disappearance of crescent-shaped dye collection. The white arrow shows the location of the previous drug-eluting stent implanted several years earlier. DES, drug-eluting stent; RCA, right coronary artery.

A 56-year-old man presented with 3 days of confusion without complaint of chest pain. Electrocardiogram (ECG) in emergency department as part of routine evaluation showed inferior ST-segment elevation. Blood pressure was 131/58 mmHg and heart rate 69 b.p.m. Cardiovascular exam was unremarkable. Except for confusion without a focal neurological deficit, physical examination was unremarkable. High sensitivity troponin was elevated at 1548 ng/L (normal < 14 ng/L). Patient had chronic kidney disease on haemodialysis and coronary artery disease with previous drug-eluting stent (DES) to the distal RCA implanted several years earlier. The patient was active without functional limitation. The patient was chronically taking nifedipine 60 mg daily, pantoprazole 20 mg daily, sevelamer carbonate 800 mg three times daily, and cinacalcet 30 mg daily. Despite the patient’s presentation being unusual for ST-elevation myocardial infarction (STEMI), he underwent emergent catheterization based on ECG, which showed 70% mid-left anterior descending and 80% distal circumflex stenoses with Thrombolysis in Myocardial Infarction (TIMI) 3 flow and 99% ostial RCA lesion with TIMI 3 flow with a very proximal crescent-shaped contrast collection with flow suggestive, but not definitive for, a communication with the extravascular space and possibly the aortic root (*Figure 1A*, see Supplementary material online, *Video S1*).

**Figure 2:**
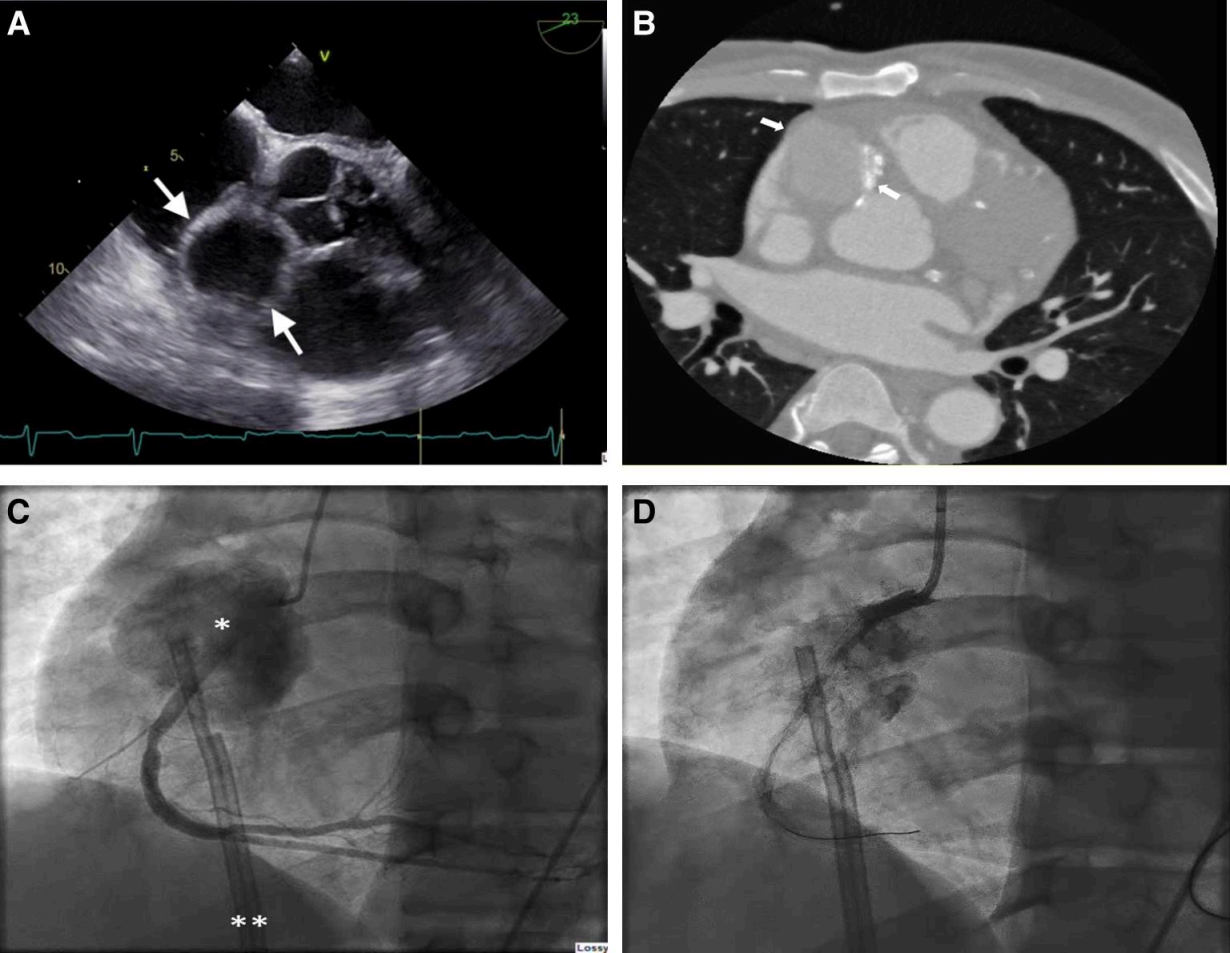
*(*A*) Transoesophageal echocardiogram on second presentation showing a large cystic structure (4.0 × 3.5 cm) on mid-oesophageal short-axis view at the aortic valve level, (*B*) computed tomography angiography showing right coronary artery pseudoaneurysm (between the arrows) abutting aortic root; the dense linear radio-opacity on the medial side of the pseudoaneurysm is a drug-eluting stent, (*C*) second presentation angiogram: left anterior oblique view of the right coronary artery showing the pseudoaneurysm labelled ‘*’ and a temporary femoral dialysis catheter labelled ‘**’, (*D*) percutaneous intervention of pseudoaneurysm: right coronary artery angiography after covered stent deployment in proximal right coronary artery. Note contrast flow distal to the covered stent into the pseudoaneurysm. DES, drug-eluting stent; RCA, right coronary artery.

Before starting the interventional procedure, the patient became hypotensive (62/33 mmHg) necessitating emergent placement of a temporary left ventricular (LV) assist device, i.e. Impella CP (Abiomed, Danvers, MA, USA). Drug-eluting stent (3 mm × 23 mm) was then implanted with disappearance of the crescent-shaped lesion (*Figure 1B*). In view of the tenuous haemodynamic status, it was decided to defer decision regarding further revascularization. No procedure-related trauma to the vessel was observed or suspected at the conclusion of the percutaneous coronary intervention (PCI). Twelve hours later, patient became febrile; multiple blood cultures showed *Staphylococcus aureus*. Transoesophageal echocardiogram (TEE) suggested a small (8 mm) mitral valve vegetation with normal LV ejection fraction and moderate mitral regurgitation. Cardiac surgeon recommended antibiotic therapy and operating only if mitral regurgitation worsened or antibiotic therapy was ineffective. Haemodynamic stability ensued; LV assist device was removed after 5 days. Patient was transferred to a rehabilitation centre 4 weeks later. Patient represented with syncope 2 weeks post-discharge with telemetry showing transient 2nd-degree heart block. Transoesophageal echocardiogram showed a large cystic structure (4 cm × 3.5 cm) near the right aortic cusp extending into the atrioventricular groove suspicious for an RCA pseudoaneurysm (*Figure 2A*, see Supplementary material online, *Video S2*); there was no evidence of mitral valve vegetation and only mild to moderate mitral regurgitation. Electrocardiogram-gated computed tomography angiography demonstrated a large cystic structure (3.5 cm × 4 cm × 4.7 cm) abutting the proximal RCA posteriorly, suspicious for a pseudoaneurysm (*Figure 2B*).

The cardiac surgical team felt patient was at high surgical risk. Heart Team recommended covered stent implantation. Angiography showed a giant RCA pseudoaneurysm (*Figure 2C*, see Supplementary material online, *Video S3*). With significant difficulty, a guidewire was passed to the distal RCA. Papyrus 4 mm × 20 mm covered stent (Biotronik AG, Bülach, Switzerland) was advanced but would not pass beyond the proximal RCA. Due to difficulty in passing wire distally, it was elected to deploy covered stent in the proximal/ostial location felt to be over the opening of the pseudoaneurysm. There was a small stent segment protruding into the aorta. Post-covered stent implantation angiography showed well-expanded stent with contrast free flowing into the pseudoaneurysm distal to the stent (*Figure 2D*, see Supplementary material online, *Video S3B*). Attempts to advance various diameter balloons into the mid-RCA were unsuccessful. Use of intravascular ultrasound was discussed but not attempted given difficulty passing hardware. It was concluded that the guidewire had passed through a stent strut of the initial DES implanted during STEMI. With the first guidewire in place, a second guidewire was passed into the distal RCA. Over the second guidewire multiple balloons were passed but also would not cross past the proximal RCA. The case was terminated for futility and patient referred for surgery.

**Figure 3:**
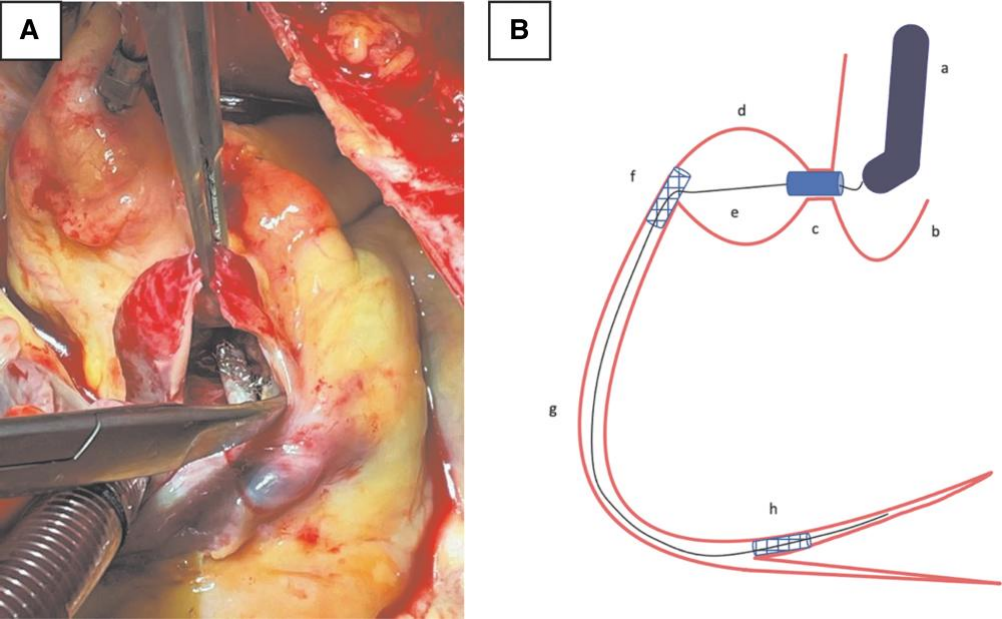
(*A*) Intraoperative view of the opened pseudoaneurysm showing covered stent and drug-eluting stent, (*B*) a schematic of the hypothesized position of the deployed covered stent and the coronary guidewire. The wire was hypothesized to pass through a stent strut preventing any catheters to traverse distally. Frequent passes of balloon catheters before and after covered stent placement caused right coronary artery transection at pseudoaneurysm site. (*A*) guide catheter, (*B*) aortic root, (*C*) covered stent, (*D*) coronary pseudoaneurysm, (*E*) coronary guidewire, (*F*) recently placed drug-eluting stent, (*G*) mid-right coronary artery, (*H*) old posterolateral branch drug-eluting stent. DES, drug-eluting stent; RCA, right coronary artery.

Intraoperatively, the vessel segment between proximal and mid-RCA was found to be transected (*Figure 3A*). The covered stent’s distal end was free-floating in the pseudoaneurysm; the proximal DES segment was crushed to the vessel wall. It was hypothesized the RCA had been transected during PCI. Patient underwent pseudoaneurysm resection, stent removal, RCA closing, and saphenous vein grafting to the posterior descending artery. Patient was discharged 2 weeks later with negative blood cultures and a repeat TEE that did not reveal any mitral valve vegetations. On 3-month follow-up, the patient was ambulatory and asymptomatic.

## Discussion

In a comprehensive review, our group classified pseudoaneurysm size relative to the originating vessel: ‘small’ (pseudoaneurysm diameter less than parent vessel), ‘moderate’ (1–2 times vessel diameter), ‘large’ (3–5 times vessel diameter), and ‘giant’ (>5 times vessel diameter)^1^as seen in this case.

The present case of giant pseudoaneurysm had some unusual aspects. On initial presentation, symptoms were not suggestive of STEMI. In retrospect, the patient may have had a septic embolus causing near RCA occlusion and a mycotic aneurysm in setting of bacteraemia causing hypotension^2^. The crescent-shaped dye stain may have represented a leak from incipient vessel perforation. That it disappeared after stenting may have been due to compressed overlying tissue temporarily occluding the leak. This scenario is conjectural but would explain initial observations. Procedure-related vessel trauma is another possibility, albeit less likely because the crescent-shaped lesion seen was present on the very first contrast injection.

Patient presented 6 weeks later with a giant aneurysm with its mouth in the same place as the initial crescent-shaped dye collection. There was TIMI 3 flow in the distal RCA so excluding the pseudoaneurysm site with a covered stent seemed appropriate. However, despite the ability to pass guidewires into distal RCA there was an inability to pass either balloon catheter or covered stent past the proximal RCA. We hypothesize the difficulty was because the coronary guide wire traversed a strut of the previously implanted DES stent (*Figure 3B*). Right coronary artery transection found at surgery may have occurred spontaneously and death averted by the pseudoaneurysm or more likely during covered stent implantation with repeated vigorous attempts to pass a balloon catheter into the mid-vessel for second covered stent implantation. In this case, a ‘Heart Team’ approach had been used; we recommend it as routine for management of a giant aneurysm, which routinely requires some type of intervention. Further, the technical issues during PCI despite the angiographic appearance of an ‘easy’ procedure illustrate the potential challenges of treating a pseudoaneurysm.

In retrospect, use of intravascular imaging during the initial STEMI PCI and prior to the attempt at covered stent implantation may have provided useful information in planning the interventions. At the end of the primary PCI, the RCA ostium was smaller than the stented area. This fact was appreciated with the thought that the ostium was ‘large enough’ at a time of tenuous haemodynamic status but intravascular imaging would have provided further information on the state of the ostium as well as clarify if a true vessel wall break was present. Prior to the subsequent attempted covered stent implantation, intravascular imaging would likely have been challenging in view of the inability to pass even the smallest balloon past the mid-RCA but its use may still have been revealing in some details of the anatomy.

Managing pseudoaneurysms appropriately are daunting in view of lack of robust evidence and guidelines. Literature review and this case emphasize that each pseudoaneurysm should be carefully assessed and management individualized. While infectious PCI complications are rare, it seems reasonable to defer stent implantation in patients with bacteraemia and limit PCI to balloon angioplasty if feasible. Because it is unlikely that a prospective case series will be forthcoming, each case report can provide further insights into treating this condition.

In conclusion, coronary artery pseudoaneurysm is a serious, potentially fatal, PCI complication. It confers a management challenge. Individualized strategies utilizing a Heart Team approach should be employed. In cases of active bacteraemia, deferring stent placement if feasible during STEMI seems appropriate after re-establishing blood flow with balloon angioplasty.

## Supplementary Material

ytad637_Supplementary_Data

## Data Availability

The data are subject to restrictions due to patient confidentiality and privacy considerations, although the data will be shared on reasonable request to the corresponding author.

## Lead author biography

Dr Alturkmani is an interventional cardiology fellow at the University of Arkansas for Medical Sciences and will be pursuing training in interventional cardiology.

## Supplementary material

**Consent**: The patient’s next of kin hereby affirms being well informed and acknowledges receiving a document acknowledging promulgation of the case report with anonymized data. The authors confirm that written informed consent for submission and publication of this case report including images and associated text has been obtained from the patient’s next of kin in line with Committee on Publication Ethics (COPE) guidance. Consent to publish was obtained from patient’s next of kin.

**Funding:** None declared.
